# Maternal glycemic parameters and adverse pregnancy outcomes among high-risk pregnant women

**DOI:** 10.1136/bmjdrc-2019-000774

**Published:** 2019-11-13

**Authors:** Yanwei Zheng, Yun Shen, Susu Jiang, Xiaojing Ma, Jiangshan Hu, Changbin Li, Yajuan Huang, Yincheng Teng, Yuqian Bao, Jian Zhou, Gang Hu, Minfang Tao

**Affiliations:** 1 Department of Obstetric and Gynaecology, Shanghai Jiao Tong University Affiliated Sixth People’s Hospital, Shanghai, China; 2 Chronic Disease Epidemiology, Pennington Biomedical Research Center, Baton Rouge, Louisiana, USA; 3 Department of Endocrinology and Metabolism, Shanghai Clinical Center for Diabetes, Shanghai Diabetes Institute, Shanghai Jiao Tong University Affiliated Sixth People’s Hospital, Shanghai, China

**Keywords:** gestational diabetes mellitus

## Abstract

**Objective:**

We aimed to investigate the association between maternal glycemic parameters and adverse pregnancy outcomes among high-risk pregnant women.

**Research design and methods:**

A total of 1976 high-risk pregnant women were enrolled between 2015 and 2017. All participants received a 75 g oral glucose tolerance test during the 24–30 gestational weeks and complete birth and delivery information was collected. Adverse pregnancy outcomes were defined as premature birth, birth weight >90th percentile, primary cesarean section, and pre-eclampsia. Logistic regression models were used to assess the association between five maternal glycemic parameters during pregnancy (fasting glucose, 1-hour glucose, 2-hour glucose, HbA1c, and serum 1,5-anhydroglucitol (1,5-AG)) and adverse pregnancy outcomes.

**Results:**

Of 1976 participants, 498 were diagnosed with gestational diabetes. The multivariable-adjusted ORs of adverse pregnancy outcomes for each one unit increase (1 mmol/L, 1%, or 1 µg/mL) were 2.32 (95% CI 1.85 to 2.92) for fasting glucose, 1.07 (95% CI 1.01 to 1.15) for 1-hour glucose, 1.03 (95% CI 0.96 to 1.10) for 2-hour glucose, 1.77 (95% CI 1.34 to 2.33) for HbA1c, and 0.96 (95% CI 0.94 to 0.98) for 1,5-AG, respectively. When all five glycemic parameters were simultaneously entered into the multivariable-adjusted model, only fasting glucose was significantly associated with total and individual adverse pregnancy outcomes. Receiver operating characteristic curve showed that fasting glucose plus any one of other four glycemic parameters had significantly enhanced the sensitivity of detecting adverse pregnancy outcomes.

**Conclusions:**

Fasting glucose at 24–30 gestational weeks was strongly associated with adverse pregnancy outcomes. Fasting glucose combined with one additional glycemic measurement showed non-inferiority indicating that post-load glycemic measurement was not necessary in detecting adverse pregnancy outcomes among high-risk pregnant women.

**Significance of this study**

**What is already known about this subject?**

- Maternal hyperglycemia is often associated with adverse pregnancy outcomes such as pre-eclampsia, macrosomia, neonatal hypoglycemia, respiratory distress syndrome, and preterm birth.

**What are the new findings?**

- Fasting plasma glucose (FPG) was a better predictor for adverse pregnancy outcomes than other glycemic parameters.
- FPG combined with one additional glycemic measurement, especially HbA1c or serum 1,5-anhydroglucitol measurement, would definitely enhance the power for detecting adverse pregnancy outcomes.
- Post-load glucose was not a good predictor for adverse pregnancy outcomes among high-risk pregnant women in Shanghai.

**How might these results change the focus of research or clinical practice?**

- Post-load glycemic measurement was not necessary in detecting adverse pregnancy outcomes among high-risk pregnant women.

## Introduction

Gestational diabetes mellitus (GDM) is defined as glucose intolerance with onset or first recognition during pregnancy.^1^ GDM is often associated with adverse pregnancy outcomes such as pre-eclampsia, macrosomia, neonatal hypoglycemia, respiratory distress syndrome, and preterm birth.^2 3^ Previous studies have shown that women with GDM have an increased risk of type 2 diabetes post partum.^4 5^ Currently, one-step oral glucose tolerance test (OGTT) is considered as a golden diagnostic criterion for GDM, which is developed by the International Association of Diabetes in Pregnancy Study Groups (IADPSG) and recommended by the American Diabetes Association and WHO.^6^ The IADPSG guideline was drafted according to the findings from the Hyperglycemia and Adverse Pregnancy Outcome (HAPO) study, which was launched in 15 sites from 9 countries.^7^ HAPO is a milestone study that links maternal hyperglycemia with adverse pregnancy outcomes. Although many countries and regions are using this criterion,^6 8^ disputes never ended in terms of the lowering of the cut-point for GDM, and the prevalence of GDM may increase doubly.^9 10^ Moreover, some researchers consider that the OGTT process is too complicated and are looking for a more simple and effective method.^11^ Glycated hemoglobin A1c (HbA1c) is more easily used as an indicator to detect changes in plasma glucose throughout pregnancy; however, HbA1c is not a substitute for OGTT diagnosis now.^12^ In recent years, serum 1,5-anhydroglucitol (1,5-AG) has been considered to be a useful glycemic marker for diabetes control and may be a new indicator for detecting GDM.^13–15^ Serum 1,5-AG reflects shorter-term changes in plasma glucose in patients compared with HbA1c.^16^ However, some studies have suggested that 1,5-AG is affected by renal excretion during pregnancy and does not relate to hyperglycemia during pregnancy. Thus, 1,5-AG is still controversial as an indicator for hyperglycemia during pregnancy.^16^

Asian women including Chinese women have a higher or same prevalence of GDM compared with whites although they have a lower body mass index (BMI) than whites.^17^ However, very few studies have assessed the association between different maternal glucose measures and adverse pregnancy outcomes among Chinese pregnant women, especially among high-risk pregnant women. Women from mainland China were not included in the HAPO study. Therefore, the aim of the present study was to investigate the association between different glycemic parameters and adverse pregnancy outcomes, and compare the most practical way of glycemic measurements for high-risk pregnant women at 24–30 gestational weeks among Chinese women.

## Research design and methods

### Shanghai critical maternal consultation and rescue center

The Department of Obstetrics and Gynecology, Shanghai Jiao Tong University affiliated Sixth People’s Hospital is one of the four critical maternal consultation rescue centers in Shanghai. The obstetric service is staffed by experienced and certified nurse midwives, nurse practitioners, and doctors. We have undertaken the task of treating maternal women with complicated high-risk pregnancy diseases in southwestern Shanghai. Therefore, a large number of pregnant women with high-risk pregnancy diseases visit here until delivery. In recent 5 years, we have successfully rescued more than 10 000 pregnant women with high-risk pregnancy diseases from all over the country and 97.1% of them completely recovered.

### Participants

We recruited all pregnant women with live births in the Department of Obstetrics and Gynecology, Shanghai Jiao Tong University affiliated to Sixth People’s Hospital in 2015–2017. Eligible participants should be over 20 years old, have complete medical and production records, and have complete records of the OGTT at 24–30 gestational weeks. Participants who were pregnant and complicated with kidney or liver disease, had multiple pregnancies, planned to deliver at another hospital, had a history of diabetes, and participated in other clinical studies during pregnancy were excluded. Among 2100 women who met the inclusion criteria, 1976 women who had complete obstetric and neonatal records and available 1,5-AG results were included in the present study.

### Anthropometric and laboratory measurements

Medical records were obtained by a blinded doctor, including information about medical history of participants and current diseases. Height and weight were measured at their first visit and BMI was calculated as weight/height^2^ (kg/m^2^). Participants underwent a 75 g OGTT on a morning fasting between 24 and 30 weeks of pregnancy. Five glycemic parameters including fasting plasma glucose (FPG), 1-hour post-load glucose (1hPG), 2-hour post-load glucose (2hPG), serum 1,5-AG, and HbA1c were measured among all participants. Plasma glucose levels were obtained by the glucose oxidase method (Kehua China Shanghai Bioengineering) using the Charisma 2000 biochemical automatic analyzer. Serum 1,5-AG was measured by an enzymatic method (GlycoMark; GlycoMark Inc., New York, New York, USA) on the 7601-120 autoanalyzer (Hitachi, Tokyo, Japan) and inter-assay and intra-assay coefficients of variation (CV) were 1.54%–3.03% and 0.83%–2.44%, respectively. HbA1c was measured by high-pressure liquid chromatography (Variant II hemoglobin analyzer; Bio-Rad, Hercules, California, USA) with inter-assay and intra-assay CVs of 0.75%–3.39% and 0.55%–2.58%, respectively.

### Diagnostic criteria

GDM was diagnosed according to the 2010 IADPSG criteria (FPG ≥5.1 mmol/L; or 1-hour post OGTT glucose ≥11.1 mmol/L; or 2-hour post OGTT glucose ≥8.5 mmol/L).^6^

### Outcomes

Adverse pregnancy outcomes were defined as birth weight >90th percentile (a newborn was considered to have a birth weight >90th percentile, if birth weight was greater than the estimated 90th percentile for the baby’s sex, gestational age, ethnicity, and maternal parity), primary cesarean section (if this delivery was the first time and was a cesarean section), pre-eclampsia (systolic blood pressure ≥140 mm Hg and/or diastolic blood pressure ≥90 mm Hg on two or more occasions with at least 6 hours apart and proteinuria ≥1+ on dipstick or ≥300 mg on 24-hour urine collection), and preterm delivery (delivery less than 37 weeks of pregnancy). Apgar score was used to determine the presence or absence of neonatal asphyxia and asphyxia, which was based on heart rate, respiration, muscle tone, laryngeal reflex, and skin color within 1 min after birth.

### Statistical analysis

The general characteristics (continuous and categorical variables) of both mothers and children according to maternal GDM status were determined using the χ^2^ test or Student t-test. Logistic regression models were used to assess the association between different glycemic parameters at 24–30 gestational weeks and adverse pregnancy outcomes. All analyses were adjusted for age (model 1), and then for BMI, family history of diabetes, gestational complications, gestational age at OGTT, infant’s sex, parities, FPG, and 2hPG (model 2). Serum cut-off values and their corresponding sensitivity and specificity for FPG, FPG+1hPG, FPG+2 hPG, FPG+HbA1c, and FPG+1,5-AG in the identification of adverse pregnancy outcomes were analyzed by the receiver operating characteristic (ROC) curve analysis. The areas under the ROC curves (AUC) were compared by the pairwise comparison analysis. A p value <0.05 was considered statistically significant. All statistical analyses were performed by IBM SPSS Statistics for Windows, V.23.0.

## Results

Of 1976 participants, 498 were diagnosed with GDM. The baseline characteristics of the participants are listed in table 1. The average age of all participants was 30.7±4.29 years. Women with GDM were older; had a higher BMI at 24–30 gestational weeks; a higher FPG, 1hPG, 2hPG, 1,5-AG, and HbA1c; and were more postpartum hemorrhage compared with women without GDM. Offspring of mothers with GDM had a higher birth weight, shorter gestational age at delivery, and had a higher prevalence of adverse pregnancy outcomes including preterm delivery, macrosomia, primary cesarean section, and pre-eclampsia than children of mothers without GDM.

**Table 1:** Characteristics of the study participants

Variables	All participants (n=1976)	Non-GDM (n=1478)	GDM (n=498)	P value
*Maternal characteristics*				
Age, years	30.7±4.29	30.5±4.21	31.3±4.45	<0.001
Body mass index at 24–28 weeks, kg/m^2^	27.4±2.73	27.2±2.57	28.1±3.07	<0.001
No of pregnancies, %				0.981
1	47.5	48.6	44.2	
2	26.3	26.3	26.3	
≥3	26.2	25.1	29.5	
Parity, %				0.544
0	68.8	69.3	67.5	
1	29.3	28.9	30.3	
≥2	1.9	1.8	2.2	
FPG, mmol/L	4.41±0.58	4.33±0.52	4.64±0.67	<0.001
1hPG, mmol/L	8.28±1.70	8.15±1.65	8.63±1.80	<0.001
2hPG, mmol/L	6.80±1.76	6.68±1.70	7.12±1.88	<0.001
HbA1c, %	5.0±0.4	5.0±0.4	5.1±0.5	<0.001
1.5-AG, µg/mL	1.78±1.00	1.90±1.00	1.42±0.90	0.002
Postpartum hemorrhage, mL	324±181	318±144	343±258	0.006
*Newborn*				
Birth weight, g	3373±553	3336±328	3479±923	<0.001
Birth length, cm	49.8±1.35	49.8±0.51	49.7±2.49	0.033
Gestational age at delivery, weeks	39.0±1.71	39.1±1.63	38.7±1.89	<0.001
Apgar score	9.96±0.37	9.96±0.37	9.95±0.38	0.722
Boys, %	53.4	53.3	53.8	0.847
Preterm delivery, %	10.2	8.53	15.3	<0.001
Birth weight >90th percentile, %	14.1	12.0	20.3	<0.001
*Obstetric outcomes*				
Primary cesarean section, %	29.8	28.5	33.5	0.036
Pre-eclampsia, %	2.9	2.4	4.4	0.016

FPG, fasting plasma glucose; GDM, gestational diabetes mellitus.

The multivariable-adjusted (age, BMI, family history of diabetes, gestational complications, gestational age at OGTT, infant’s sex and parities) ORs of adverse pregnancy outcomes across quartiles of FPG were 1.00, 1.19, 1.40, and 2.57 (p for trend <0.001), respectively (table 2). The multivariable-adjusted positive associations were found when 1hPG, HbA1c, and 1,5-AG were used as independent variables. However, the associations of 2hPG with adverse pregnancy outcomes were not significant after multivariable adjustments.

**Table 2:** ORs of adverse pregnancy outcomes by different glycemic markers

	Quartile 1	Quartile 2	Quartile 3	Quartile 4	P for trend	As continuous variables
FPG						
No of patients	502	494	494	486		
No of cases	182	194	217	296		
Model 1	1.00	1.13 (0.88–1.46)	1.37 (1.06–1.77)	2.70 (2.89–3.50)	<0.001	2.40 (1.96–3.95)
Model 2	1.00	1.19 (0.92–1.55)	1.40 (1.08–1.81)	2.57 (1.95–3.38)	<0.001	2.32 (1.85–2.92)
1hPG						
No of patients	495	497	491	493		
No of cases	191	218	231	249		
Model 1	1.00	1.24 (0.96–1.60)	1.40 (1.09–1.80)	1.59 (1.23–2.05)	0.004	1.13 (1.07–1.20)
Model 2	1.00	1.23 (0.95–1.59)	1.28 (0.99–1.67)	1.12 (0.81–1.55)	0.235	1.07 (1.01–1.15)
2hPG						
No of patients	499	494	490	493		
No of cases	203	206	227	253		
Model 1	1.00	1.04 (0.81–1.34)	1.24 (0.96–1.60)	1.50 (1.17–1.94)	0.006	1.11 (1.05–1.17)
Model 2	1.00	1.01 (0.78–1.30)	1.17 (0.91–1.52)	1.05 (0.76–1.45)	0.591	1.03 (0.96–1.10)
HbA1c						
No of patients	609	517	443	407		
No of cases	248	216	192	233		
Model 1	1.00	1.04 (0.82–1.32)	1.10 (0.86–1.41)	1.91 (1.48–2.47)	<0.001	1.99 (1.53–2.59)
Model 2	1.00	1.03 (0.81–1.32)	1.07 (0.83–1.38)	1.72 (1.31–2.24)	<0.001	1.77 (1.34–2.33)
1,5-AG						
No of patients	518	486	499	473		
No of cases	284	213	205	187		
Model 1	1.00	0.65 (0.51–0.84)	0.58 (0.45–0.74)	0.55 (0.42–0.70)	<0.001	0.95 (0.93–0.97)
Model 2	1.00	0.67 (0.52–0.86)	0.63 (0.49–0.81)	0.62 (0.47–0.81)	<0.001	0.96 (0.94–0.98)

Model 1 adjusted for age.

Model 2 adjusted for age, body mass index, family history of diabetes, gestational complications, gestational age at oral glucose tolerance test, infant’s sex, and parities.

FPG, fasting plasma glucose.

When five glycemic parameters were considered as continuous variables, the multivariable-adjusted ORs of adverse pregnancy outcomes were 2.32 (95% CI 1.85 to 2.92) for each 1 mmol/L increase in FPG, 1.07 (95% CI 1.01 to 1.15) for each 1 mmol/L increase in 1hPG, 1.03 (95% CI 0.96 to 1.10) for each 1 mmol/L increase in 2hPG, 1.77 (95% CI 1.34 to 2.33) for each 1% increase in HbA1c, and 0.96 (95% CI 0.94 to 0.98) for each 1 µg/mL increase in 1,5-AG, respectively.

When all five glycemic parameters were simultaneously entered into the multivariable-adjusted model, only FPG (OR 2.16; 95% CI 1.70 to 2.74) and 1,5-AG (OR 0.97; 95% CI 0.94 to 0.99) were significantly associated with adverse pregnancy outcomes (table 3). FPG was also significantly associated with the risks of preterm delivery (OR 1.55; 95% 1.11 to 2.16), birth weight >90th percentile (OR 2.79; 95% CI 2.02 to 3.83), and primary cesarean section (OR 1.34; 95% 1.05 to 1.72), but not pre-eclampsia (OR 0.61; 95% 0.29 to 1.26). HbA1c was significantly associated with the risk of pre-eclampsia (OR 5.38; 95% CI 2.19 to 13.2). No associations of 1hPG, 2hPG, HbA1c, and 1,5-AG with other adverse pregnancy outcomes were found.

**Table 3:** ORs of total and individual adverse pregnancy outcome by different combinations of glycemic markers

	Glycemic markers	All outcomes	Preterm delivery	Birth weight >90th percentile	Primary cesarean section	Pre-eclampsia
1	FPG	2.32 (1.86–2.90)	1.62 (1.19–2.20)	3.09 (2.29–4.16)	1.39 (1.11–1.75)	0.97 (0.51–1.87)
	1hPG	1.03 (0.97–1.10)	1.00 (0.89–1.12)	1.03 (0.93–1.14)	1.00 (0.92–1.08)	1.17 (0.93–1.47)
2	FPG	2.35 (1.89–2.92)	1.78 (1.33–2.36)	2.95 (2.26–3.85)	1.43 (1.16–1.77)	1.23 (0.68–2.24)
	2hPG	1.03 (0.97–1.09)	1.03 (0.94–1.13)	1.02 (0.95–1.11)	0.99 (0.94–1.06)	1.11 (0.93–1.34)
3	FPG	2.25 (1.80–2.82)	1.75 (1.29–2.37)	2.69 (2.03–3.56)	1.40 (1.12–1.75)	0.80 (0.42–1.50)
	HbA1c	1.28 (0.95–1.72)	1.16 (0.74–1.810)	1.45 (0.97–2.16)	1.07 (0.78–1.47)	5.08 (2.18–11.8)
4	FPG	2.32 (1.87–2.86)	1.80 (1.37–2.35)	2.93 (2.28–3.79)	1.37 (1.12–1.68)	1.49 (0.86–2.59)
	1,5-AG	0.97 (0.94–0.99)	0.98 (0.95–1.02)	0.98 (0.94–1.01)	0.97 (0.94–0.99)	1.02 (0.95–1.09)
5	FPG	2.32 (1.85–2.90)	1.61 (1.19–2.20)	3.10 (2.30–4.18)	1.39 (1.10–1.75)	0.97 (0.50–1.88)
	1hPG	1.02 (0.94–1.10)	1.01 (0.89–1.15)	1.01 (0.90–1.13)	1.00 (0.92–1.10)	1.20 (0.92–1.56)
	2hPG	1.02 (0.95–1.10)	0.98 (0.87–1.10)	1.04 (0.94–1.16)	0.98 (0.90–1.07)	0.95 (0.74–1.22)
6	FPG	2.19 (1.73–2.77)	1.55 (1.11–2.17)	2.80 (2.04–3.86)	1.36 (1.06–1.74)	0.59 (0.28–1.22)
	1hPG	1.02 (0.94–1.10)	1.01 (0.89–1.15)	1.00 (0.89–1.12)	1.00 (0.92–1.10)	1.19 (0.92–1.55)
	2hPG	1.01 (0.94–1.09)	0.97 (0.86–1.09)	1.03 (0.92–1.15)	0.98 (0.90–1.07)	0.87 (0.68–1.12)
	HbA1c	1.25 (0.93–1.69)	1.15 (0.73–1.82)	1.43 (0.95–2.15)	1.08 (0.78–1.49)	5.13 (2.10–12.5)
7	FPG	2.16 (1.70–2.74)	1.55 (1.11–2.16)	2.79 (2.02–3.83)	1.34 (1.05–1.72)	0.61 (0.29–1.26)
	1hPG	1.01 (0.94–1.10)	1.01 (0.89–1.14)	1.00 (0.89–1.12)	1.00 (0.92–1.09)	1.19 (0.91–1.55)
	2hPG	0.99 (0.93–1.08)	0.97 (0.86–1.09)	1.02 (0.92–1.14)	0.97 (0.89–1.05)	0.89 (0.69–1.15)
	HbA1c	1.21 (0.90–1.64)	1.14 (0.72–1.80)	1.39 (0.92–2.10)	1.04 (0.76–1.44)	5.38 (2.19–13.2)
	1,5-AG	0.97 (0.94–0.99)	0.99 (0.95–1.03)	0.98 (0.95–1.01)	0.97 (0.94–0.99)	1.05 (0.97–1.13)

All analyses adjusted for age, body mass index, family history of diabetes, gestational complications, gestational age at oral glucose tolerance test, infant’s sex and parities, as well as all glycemic markers included in the combinations.

FPG, fasting plasma glucose.

**Figure 1:**
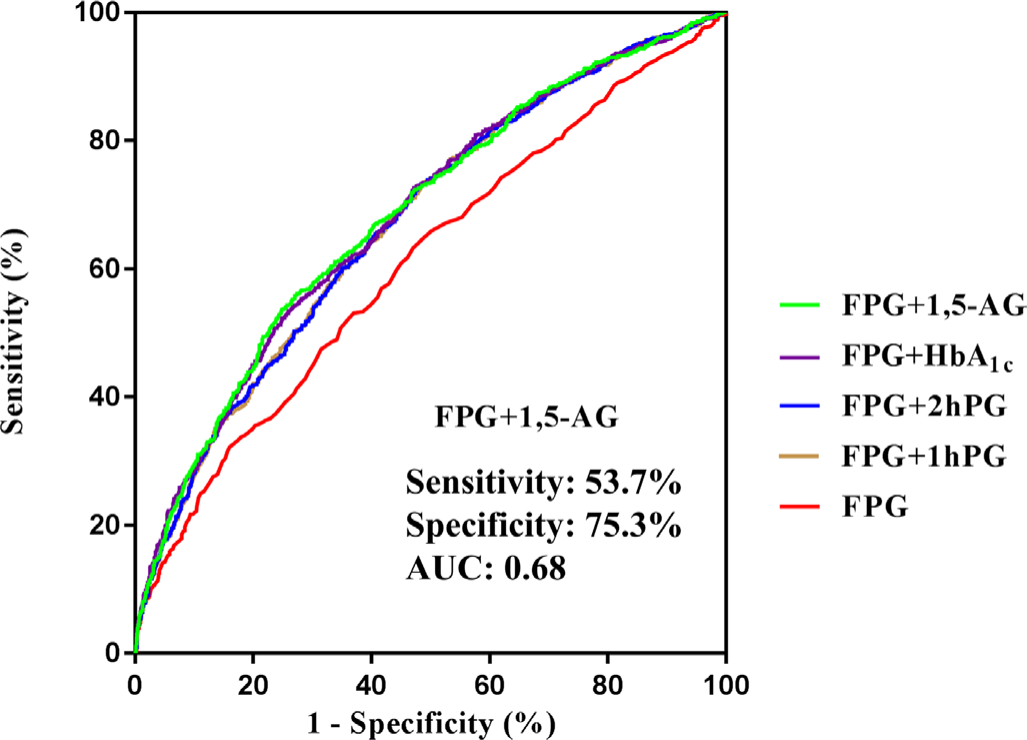
ROC curves for the identification of adverse pregnancy outcomes by FPG, and FPG combined with any one 1hPG, 2hPG, HbA1c, and 1,5-AG. AUC, area under the curve; FPG, fasting plasma glucose; HbA1c, glycated hemoglobin A1c; ROC, receiver operating characteristic.

The optimal cut-point for FPG in reflecting adverse pregnancy outcomes was 4.72 with a low sensitivity of 32.2% and a high specificity of 84.0% when using the ROC curves (figure 1). The corresponding AUC was 0.61 (95% CI 0.58 to 0.63) with p value <0.001. In order to increase the sensitivity of FPG, we combined FPG with another glucose measurement including FPG+1hPG, FPG+2hPG, FPG+HbA1c, and FPG+1,5-AG, and the response AUCs increased to 0.67 (95% CI 0.65 to 0.70), 0.67 (95% CI 0.65 to 0.70), 0.68 (95% CI 0.66 to 0.71), and 0.68 (95% CI 0.66 to 0.71), respectively. No differences among these AUCs were found by the pairwise comparison analysis. The corresponding sensitivity and specificity for these combinations were also similar.

## Discussion

In this observational study with a large sample size of high-risk pregnant women in Shanghai, we found that FPG was a better predictor for adverse pregnancy outcomes than other glycemic parameters. FPG combined with one additional glycemic measurement, especially HbA1c or 1,5-AG measurement, would definitely enhance the power for detecting adverse pregnancy outcomes. Our findings indicated that post-load glucose was not a good predictor for adverse pregnancy outcomes among high-risk pregnant women in Shanghai.

The association between hyperglycemia and adverse pregnancy outcomes is well established.^18^ The milestone HAPO multicenter study involving more than 25 000 pregnancy women suggested that increasing levels of fasting, 1-hour, and 2-hour plasma glucose were significantly related to birth weight >90th percentile and cord plasma serum C-peptide level and were less significantly related to primary cesarean delivery and neonatal hypoglycemia.^12^ From the secondary outcomes of that study, premature delivery, shoulder dystocia or birth injury, intensive neonatal care, hyperbilirubinemia, and pre-eclampsia were associated with increasing glucose levels. Our findings were consistent with the results from the HAPO study that hyperglycemia was graded and positively associated with adverse pregnancy outcomes, especially when FPG, HbA1c, and 1,5-AG were applied.

Our hospital is one of the critical maternal consultation and rescue centers in Shanghai. High-risk pregnant women were all referred to our hospital, resulting in a relatively high prevalence of adverse pregnancy outcomes in our study. Studies of hyperglycemia and adverse outcomes among these women are rare. For these high-risk pregnant women, we try to work out an optimal profile for the screening of hyperglycemia. Our results suggested that FPG had a relatively low sensitivity in identification of adverse pregnancy outcomes. Meanwhile, FPG plus an additional measurement of other glycemic parameters such as 1hPG, 2hPG, HbA1c, or 1,5-AG would definitely increase the sensitivity. However, OGTT which read 1hPG and 2hPG were always limited to the intolerance of patients and poor consistency among different patients. Our findings also showed non-inferiority among combinations of these glycemic parameters, indicating that glycemic parameters were irrespective of blood sampling and were also effective in the screening of maternal hyperglycemia.

1,5-AG has been used as a marker for studying glucose levels in recent years, especially among patients with type 2 diabetes.^19^ However, few studies on 1,5-AG were conducted in women with GDM, especially in the Chinese population. 1,5-AG, as a biomarker that does not need fasting, is not affected by glycolysis and is easily assayed. It is also a good biomarker for screening gestational diabetes in the early pregnancy.^20^ 1,5-AG was found to be well associated with neonatal birth weight in diabetic pregnancies.^21^ Our results also found a consistent inverse association of 1,5-AG with adverse pregnancy outcomes. After adjusting for other maternal glycemic parameters, 1,5-AG was no longer significant. On the other hand, 1,5-AG was not so good as FPG in the identification of adverse pregnancy outcomes. However, combination of FPG and 1,5-AG showed a similar power in identifying adverse pregnancy outcomes as other glycemic parameters. Thus, 1,5-AG can be a potential biomarker for screening GDM in early pregnancies.

The strength of our study includes the large sample size of high-risk pregnant women with a 75 g OGTT and the detailed medical records during pregnancy. There are also some limitations in our study. First, it was a single-center prospective observational study with a relatively high prevalence of adverse outcomes. Tests with high sensitivity or specificity developed in a high prevalence setting can yield low positive predictive values in the general population. Therefore, our results need to be validated by other studies with the general populations. Second, several lifestyle and socioeconomic variables including smoking, alcohol use, family income, physical activities, and so on were not obtained in this study. The effects of these factors cannot be excluded.

In conclusion, in this prospective observational study with a large sample size of high-risk pregnant women, we found that FPG was significantly associated with adverse pregnancy outcomes. FPG combined with one additional glycemic measurement would definitely enhance the power for the identification of adverse pregnancy outcomes.
